# Variant PTA Terminating in Cerebellar Artery, Associated with Multiple Aneurysms

**DOI:** 10.1155/2016/4594326

**Published:** 2016-06-30

**Authors:** Yeong Uk Hwang, Jin Woo Kim

**Affiliations:** Department of Radiology, Inje University Ilsan Paik Hospital, Goyang, Gyeonggi 10380, Republic of Korea

## Abstract

Persistent trigeminal artery (PTA) is one of the remnant fetal anastomoses between the carotid artery and basilar artery. PTAs are classified according to angiographic appearance and various connection. Among them, those directly terminating in the cerebellar arteries are rare subtype. In addition, aneurysms of the PTA are unusual in the literature and have not previously accompanied this subtype of PTA connecting cerebellar artery. We present the first case of an aneurysm of the PTA which is directly terminating in the cerebellar arteries and combined with multiple aneurysms.

## 1. Introduction

Persistent trigeminal artery (PTA) is unusual anastomosis between the carotid artery and basilar artery, with an incidence between 0.1% and 0.3% [1]. In previous studies, cases of PTA terminating in cerebellar artery are extremely rare [2, 3]. PTA can be associated with numerous anomalies in the cerebral vasculature, including aneurysm of the circle of Willis and PTA itself [4]. We describe the first case of variant PTA terminating in cerebellar artery, which is accompanied by PTA aneurysm and multiple aneurysms of the other intracranial vessels.

## 2. Case Report

A 39-year-old woman presented to an outside institution with acute onset of headache. The patient had no significant past or family medical history except major depressive disorder for 4 months. Cranial computed tomography (CT) was initially performed in the other institution and showed diffuse subarachnoid hemorrhage. She was subsequently transferred to our institution for further evaluation.

**Figure 1:**
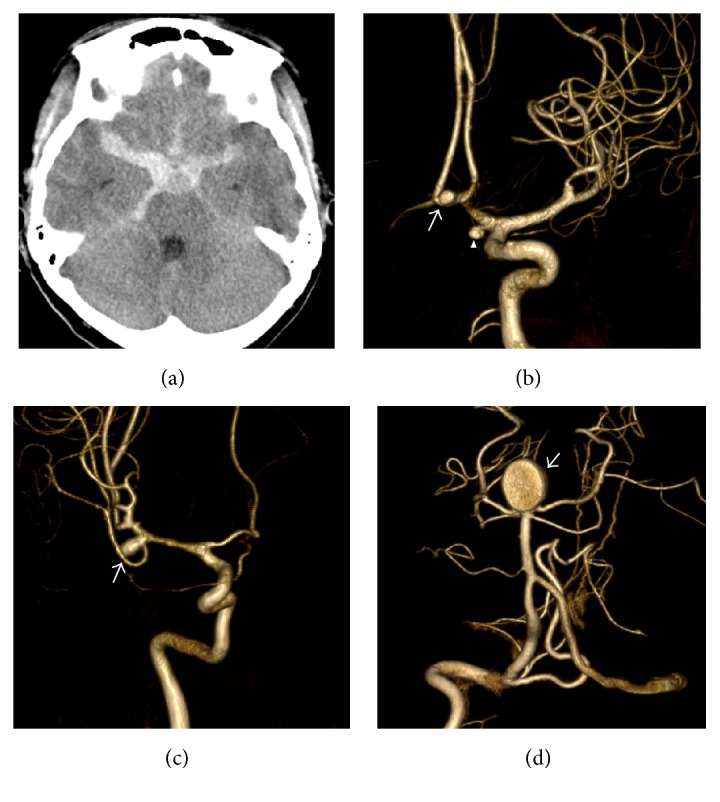
(a) Noncontrast brain CT shows diffuse subarachnoid hemorrhage. Three-dimensional digital subtraction angiography demonstrating anterior communicating artery aneurysm ((b) arrow), A1 segment of left anterior cerebral artery ((b) arrowhead), bifurcation of right middle cerebral artery aneurysm ((c) arrow), and basilar artery bifurcation aneurysm ((d) arrow).

**Figure 2:**
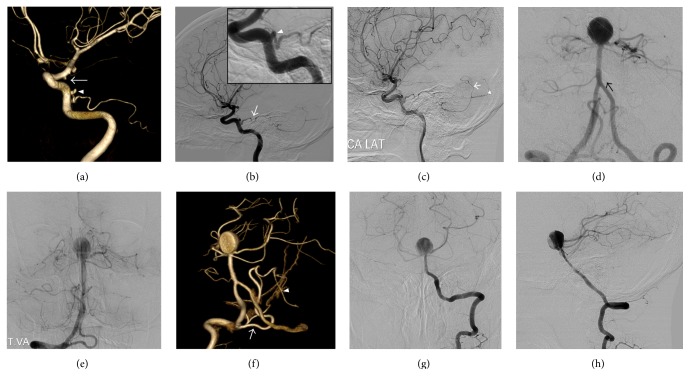
(a) Three-dimensional digital subtraction angiography shows left posterior communicating artery aneurysm (arrow) and left persistent trigeminal artery (PTA) aneurysm (arrowhead). (b) Left internal carotid injection in the lateral projection demonstrates a PTA (arrow). Inset box is a magnified view (PTA aneurysm, arrowhead). (c) Left internal carotid injection in late arterial phase showing a PTA that supplies the anterior inferior cerebellar arteries (AICA) and inferior vermian (arrow) and hemispheric (arrowhead) branches of posterior inferior cerebellar arteries (PICA). (d) Right vertebral artery injection shows left AICA hypoplasia (arrow). (e) Left vertebral injection in the early capillary phase showing the relative lack of capillary staining in the left inferolateral cerebellar hemisphere. (f) Three-dimensional digital subtraction angiography shows right PICA (arrow) crossing midline and suppling left PICA territory (arrowhead). The left vertebral angiography in the anteroposterior (g) and lateral (h) projection shows left AICA hypoplasia and left posterior inferior cerebellar artery (PICA) aplasia.

The CT-angiography (CTA) and digital subtraction angiography (DSA) showed multiple cerebral aneurysms at the site of bifurcation of right middle cerebral artery, A1 segment of left anterior cerebral artery, anterior communicating artery, left posterior communicating artery, and basilar artery bifurcation (Figure 1). Incidentally, PTA was identified arising from precavernous segment of left ICA and accompanied by PTA aneurysm itself. A PTA was connecting with left AICA and branch of PICA without joining the basilar artery (Saltzman classification III). The left vertebral angiography has shown left AICA hypoplasia and left posterior inferior cerebellar artery (PICA) aplasia. The right vertebral angiography has shown the right PICA crossing midline and then capillary filling of both cerebellar hemispheres on capillary phase, which is called bihemispheric PICA. However, there is relative lack of capillary filling in the left PICA territory at the inferolateral hemisphere. The PTA not only was terminated in the AICA but contributed to the ipsilateral inferior vermian and hemispheric branch of PICA (Figure 2). The patient had undergone clipping of multiple aneurysms (bifurcation site of right middle cerebral artery, left anterior communicating artery, and A1 segment of left anterior cerebral artery) and coiling of basilar artery bifurcation aneurysm. The PTA aneurysm was planned to have regular follow-ups. No neurological deficits developed during the treatment, and the patient was discharged from hospital without neurological deficits.

## 3. Discussion

According to embryogenesis of the carotid-basilar anastomoses described by Padget [5], there are four embryonic communicating arteries between the vertebrobasilar and carotid systems: the hypoglossal, otic, proatlantal, and trigeminal arteries. These fetal anastomoses form on approximately the 24th day of fetal embryogenesis, when the embryo is 3 mm in size [5]. With the development of the posterior communicating artery and fusion of the paired longitudinal neural arteries into the basilar artery, these anastomoses begin to regress and disappear by the 14 mm stage [5]. Failure on this mechanism explains the persistence of the carotid-vertebrobasilar anastomosis in the adult. The persistent trigeminal artery (PTA) is the most common anastomosis after birth, with reported incidence between 0.1% and 0.3% [1]. The angiographic anatomy and classification of PTA variants were first described by Saltzman [6]. Saltzman divided PTA variants into three types. The Saltzman type I PTA joins the basilar artery at the level between superior cerebellar artery (SCA) and anterior inferior cerebellar artery (AICA). The proximal basilar artery and posterior communicating artery are usually hypoplastic. The PTA supplies both posterior cerebral arteries and SCA. The Saltzman type II PTA connects the basilar artery above the origin of the SCAs. The posterior communicating arteries are present and supply the posterior cerebral arteries. The Saltzman type III PTA is considered a combination of types I and II. Ali et al. [7] reviewed subtypes of Saltzman type III. Saltzman type III PTAs arise from the internal carotid and terminate directly in the SCA (type IIIa), AICA (type IIIb), or PICA (type IIIc) without interposition of the basilar artery (Table 1). According to this classification of the PTA, the PTA of our report is consistent with Saltzman type IIIb + type IIIc.

The incidence of PTA variants (Saltzman type III) is extremely rare, approximately 0.18% on DSA and 0.76% on MR angiography [8]. In most cases, PTA and its variants have been found incidentally but can be associated with numerous anomalies in the cerebral vasculature, including arteriovenous malformation, aneurysms of the circle of Willis, carotid and vertebral artery agenesis, carotid-cavernous fistula, and moyamoya disease [3, 7]. Our patient had multiple aneurysms and, of these, one aneurysm is located on the origin of proximal trunk of the variant PTA. To our knowledge, our case is the first case of an aneurysm of Saltzman type IIIb combined with multiple aneurysms.

The pathogenesis of aneurysms associated with PTA is not well known. Bingham and Hayes [9] described that persistent embryological vessels were more predisposed to aneurysmal formation due to structural defect. Kim et al. [10] also suggested congenital defect of the PTA and hemodynamic stress by its anatomic location between two major arterial systems.

In conclusion, we present the first case of an aneurysm of the PTA which is connecting with cerebellar arteries and combined with multiple aneurysms. Although PTA and combined multiple vascular abnormalities including multiple aneurysm are rare, neuroradiologists and neurosurgeons should recognize these rare variations before surgery or endovascular intervention because their unawareness may be responsible for hemorrhage and ischemia in brain stem and cerebellum by vascular injury or uncontrolled emboli passing through these anomalous vessels.

## Figures and Tables

**Table 1:** Saltzman classification of persistent trigeminal artery.

Saltzman type	Termination site	Pcom
I	Basilar artery between the SCA and the AICA	Hypoplasia
II	Basilar artery above the origin of the SCA	Patent
IIIa	Directly the SCA	Patent
IIIb	Directly the AICA	Patent
IIIc	Directly the PICA	Patent

Pcom, posterior communicating artery; SCA, superior cerebellar artery; AICA, anterior inferior cerebellar artery; and PICA, posterior inferior cerebellar artery.
